# Clinical Progress and Optimization of Information Processing in Artificial Visual Prostheses

**DOI:** 10.3390/s22176544

**Published:** 2022-08-30

**Authors:** Jing Wang, Rongfeng Zhao, Peitong Li, Zhiqiang Fang, Qianqian Li, Yanling Han, Ruyan Zhou, Yun Zhang

**Affiliations:** 1 School of Information, Shanghai Ocean University, Shanghai 201306, China; 2 Key Laboratory of Fishery Information, Ministry of Agriculture, Shanghai 200335, China

**Keywords:** visual prosthesis, artificial vision, dropout and distorted phosphenes, computer vision, optimization strategy

## Abstract

Visual prostheses, used to assist in restoring functional vision to the visually impaired, convert captured external images into corresponding electrical stimulation patterns that are stimulated by implanted microelectrodes to induce phosphenes and eventually visual perception. Detecting and providing useful visual information to the prosthesis wearer under limited artificial vision has been an important concern in the field of visual prosthesis. Along with the development of prosthetic device design and stimulus encoding methods, researchers have explored the possibility of the application of computer vision by simulating visual perception under prosthetic vision. Effective image processing in computer vision is performed to optimize artificial visual information and improve the ability to restore various important visual functions in implant recipients, allowing them to better achieve their daily demands. This paper first reviews the recent clinical implantation of different types of visual prostheses, summarizes the artificial visual perception of implant recipients, and especially focuses on its irregularities, such as dropout and distorted phosphenes. Then, the important aspects of computer vision in the optimization of visual information processing are reviewed, and the possibilities and shortcomings of these solutions are discussed. Ultimately, the development direction and emphasis issues for improving the performance of visual prosthesis devices are summarized.

## 1. Introduction

In the 2019 World Health Organization report on global vision [1], it was revealed that at least 2.2 billion people worldwide have various vision problems, and Dr. Tedros added that 65 million people are blind or visually impaired. The main causes of blind-ness are retinal diseases, both hereditary and acquired. Among these, retinal degenerative diseases such as retinitis pigmentosa (RP) and age-related macular degeneration (AMD) are irreversible causes of blindness.

Loss of vision can cause great difficulties for humans to perform some tasks in daily life. People who are visually impaired can accomplish simple braille reading with the help of touch, some simple tasks with the help of hearing, daily walking and going out with the help of guide dogs, etc. With the development of technology, wearable and implantable visual aid electronic devices have benefited a certain number of visually impaired patients. These devices can improve partial functional vision by inducing optical illusions from implanted electrodes through techniques of artificial vision generation on the basis of a partially intact visual pathway.

Research on artificial vision generation has been conducted since the 1950s, including the 1956 discovery by the American scientist Tassiker [2] that subretinal implantation of a photosensitive selenium cell could help humans generate light perception, and since then, researchers have started to investigate electrical stimulation to elicit light perception. In 1974, Dobelle [3] implanted electrodes in the visual cortexes of 15 visually impaired patients and produced light perception. Dobelle’s clinical results gave hope to researchers and inspired them to think about the site of electrode implantation. Inspired by these studies, from 1974 to the present researchers have carried out a variety of visual aids with electronic devices at the site of implantation and called these devices for artificial vision generation visual prostheses. Depending on the implantable site of the electrode, visual prostheses can be classified into four types: retinal prostheses, choroidal prostheses, cortical prostheses, and optic nerve prostheses. Retinal prostheses that are less invasive, have lower surgical risk, and are implanted closer to the central visual area have become the focus in this field.

Research on retinal prostheses was initiated in 1989 by the Rizzo group in the United States, which was the first to investigate artificial prostheses that could help restore vision in patients blinded by RP and AMD [4]. Since 1990, the Humayun group [5] in the United States has continued to develop retinal prostheses and conducted the first clinical trial in 2002, implanting 16 electrodes into the retinas of six patients who could perceive discontinuous light perception after surgery. In order to improve the visual perception of the implants, the retinal prosthesis Argus^®^ II, with 60 electrodes, was proposed in 2007 and was approved to enter the clinical stage in 2011 [6]. Increasing the number of implanted electrodes under the limited human tolerance and providing patients with a 20° viewing angle can help some patients to complete the recognition of simple single letters of the alphabet. The hardware enhancement, although improving the vision perception, is far from satisfying the patients’ visual requirements, and there are limitations in the maximum viewing angle that can be provided. With the limitation of hardware technology enhancement, researchers started the study of image processing optimization strategies.

Image processing plays an important role in the field of ophthalmology, such as fundus lesion detection [7,8,9,10]. Optimization strategies for image processing mainly focus on performing operations such as foreground target extraction [11,12,13,14], edge detection [15,16,17,18], and segmentation [19,20,21,22] on images captured by visual prostheses using relevant methods of computer vision or perform corrections on the generated optical illusion arrays. Early works paid attention to enhance features of images or objects. In 2008, Boyle et al. [23] used a method with an enlarged window for saliency detection to focus on human facial features inside the window to help subjects complete face recognition. With the improvement in computer vision technology, object detection and instance segmentation are being applied more in this field. Chris et al. [24] proposed an adaptive enhancement model that performs the local attenuation of less important regions and ensures the prominence of important features to enhance perception in low resolutions. Some studies have tried to improve the image acquisition method, such as Li et al. [25], who captured outside images with a depth camera and separated the object or person from the background with a segmentation model. To deal with the phenomenon of the visual irregularity of prostheses, some researchers have proposed certain image correction methods, and this field of work is more focused on Chinese character recognition, such as in [26]. Image processing techniques have improved the limited artificial visual perception induced by visual prostheses to some extent, and how to appropriately apply them to visual prosthesis device performance enhancement is the focus of current researchers’ attention.

This paper first reviews the recent clinical implantation of different types of visual prostheses, summarizes the artificial visual perception of implant recipients, and especially focuses on its irregularities, such as dropout and distorted phosphenes.

This paper summarizes the clinical progress and the visual perception of visual prosthesis implant recipients in recent years, followed by an overview of visual information optimization studies for prosthetic devices, and then discusses the possibilities and shortcomings of analyzing these optimization strategies.

## 2. Clinical Advances in Visual Prosthetics

Visual prosthesis research has been conducted for 50 years since the 1970s, and some prostheses have been approved for the clinical phase. Different types of visual prostheses require different surgical procedures, and the corresponding clinical trial data vary. Information on the implantation site, the number of implanted electrodes, the clinical trial number, and the status of the different visual prostheses in recent years is summarized in Table 1 [27]. In addition, the clinical trial reports are analyzed and summarized for some typical prosthetic devices, i.e., the real rehabilitation of visual function and the vision perception of patients after implantation.

**Table 1:** Progress on clinical trials of visual prosthetics.

Implant Site	Visual Prosthesis	Electrode Number	Visual Implant Vision	Clinical Trial Numbers	Status
Epiretinal	Argus^®^ II [5,6,28,29,30,31,32,33,34,35,36,37,38,39]	60	20/1260	NCT03635645	Received the CE mark in 2011, FDA approval in 2013, and two patients to identify a subset of the Sloan letters.
	IRIS II [40]	150	NA	NCT02670980	Ten patients evaluated for functional visual tasks for up to 3 years
	IMI [41,42]	49	NA	NCT02982811	Follow-up of 20 patients with faint light perception for 3 months
Subretinal	Alpha-AMS [43,44,45]	1500	20/546	NCT03629899	Received the CE mark in 2013 and had patients achieve an optimal visual acuity of 20/546.
	PRIMA [46,47,48,49,50,51]	378	20/460	NCT03333954	Implantation of PRIMA to five patients was started in 2017 with 36 months of follow-up.
	Suprachoroidal retinal prosthesis [52,53]	49	NA	NCT05158049	Seven implants were assessed for vision, orientation, and movement.
	Bionic Eye [54,55,56,57,58]	44	NA	NCT03406416	The safety of the device was evaluated in 2018 by implantation in four subjects with increased electrode–retinal distance and stable impedance after the procedure, with no side effects.
Intracortical	ORION [59]	60	NA	NCT03344848	Six patients without photoreceptors were approved by the FDA to be implanted in 2017, and each implant recipient received a 5-year follow-up; data from the relevant trials are not yet publicly available.
optic cortex	ICVP [59,60,61]	144	NA	NCT04634383	Five participants, tested weekly for 1 to 3 years, were assessed for electrical-stimulation-induced visual perception.
	CORTIVIS [18,59,62]	100	NA	NCT02983370	After receiving FDA approval, it was implanted in five patients for six months in 2019.

Data source: National Center for Biotechnology Information official website.

### 2.1. Epiretinal Prostheses

**Figure 1:**
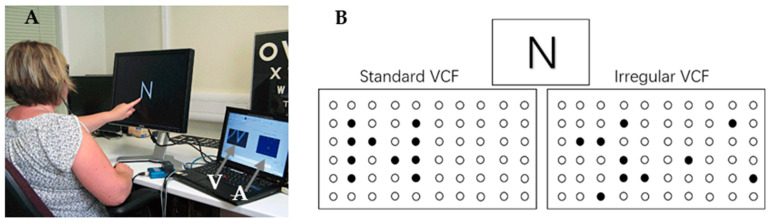
(**A**) shows an individual letter recognition task (dark environment). The display shows the letters in white in Century Gothic font on a black background, and the monitor next to it shows the camera view (V) and array (A) in real time; (**B**) is an illustration of the difference between the electrode activation maps in standard and scrambled modes when the camera is viewing the letter “N”. The correspondence between the real position of the phosphenes and the stimulus position on the array were randomized in the scrambled mode (Adapted with permission from Ref. [62]. Copyright 2011 Royal Australian and New Zealand College of Ophthalmologists).

**Figure 2:**
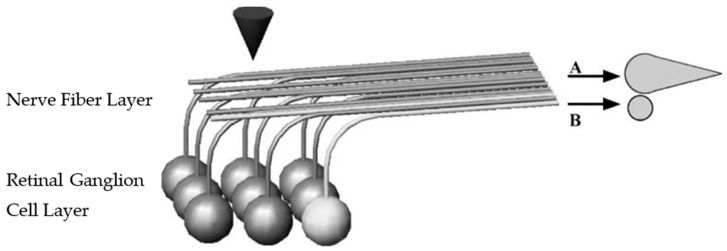
Retinal ganglion cells with 90-degree axonal curvature. (A) Diffuse streaky phosphenes produced by stimulation of distant retinal ganglion cells through the overlying axons. (B) Direct stimulation of retinal ganglion cells beneath electrodes produces punctuate phosphene (cited from [34]).

The study of the Argus series of retinal prostheses began in 1990, which achieved rapid development and gained more attention, with the first implantation into patients and long-term trials in 2002 in a total of six patients with end-stage RP. The first-generation device, Argus^®^ I, based on the cochlear implant, was implanted into the patient’s eye with a 4 × 4 array of electrodes and carried out several visual tasks [6]: locating a white square on a black background, pointing out the path of a white line above a black background, and finding a door in a room. The results of the clinical trial showed that patients could recognize simple geometric shapes with the help of the prosthesis, and one patient showed some improvement in visual perception during the target localization and mobility tests. In addition to using white square location tests, Dagnelie et al. [28] attempted to have implants perform sock classification (black and white only) and showed that the average success rate for sock classification in subjects with 60 electrodes was around 50%. With the same electrodes, the Humayun group implanted a permanent retinal prosthesis in the eye of a completely blind patient and performed individual letter recognition and word reading tests, showing that the patient was able to correctly recognize individual letters within 6 s to 221 s. During the test, the patient indicated that not all electrodes worked properly [32] and that the phosphene array, seen via electrode stimulation, had large distortions and some degree of loss of visual information, which may increase the difficulty to perform character recognition. A schematic representation of the phenomenon of dropout or distorted individual letters during recognition is shown in Figure 1. The same feedback of visual information loss was obtained from other patients implanted with this prototype device under the same circumstances [5,6,33]. With a limited number of implanted electrodes, implanters could only perform some simple visual tasks such as letter recognition. Meanwhile, it was found that the patients saw a lower resolution than the number of implanted electrodes, with a distorted array of electrodes after surgery. This may have been due to the implanted electrodes not working or because the electrodes were implanted in necrotic tissues from simulation experiments [34]. The principle of inducing dropout or distorted phosphene is illustrated in Figure 2.

The second generation of the Argus product, Argus^®^ II, increases the number of implanted electrodes to a 6 × 10 array and installs a camera on the device’s eyeglasses that captures images, which are processed and coded to transmit stimulus commands to the electrode array, producing corresponding phosphenes. Not only does the second-generation product have an increased number of electrodes and theoretically evoke better visual information from electrodes, the Argus^®^ II product was the first visual prosthesis in the world to receive CE mark approval and FDA approval, making the Argus^®^ II among the most common implants worldwide [35]. Breanne et al. [36] used the second-generation product to test patients for single-letter recognition, where the test letters were a subset of the Sloan alphabet containing O, V, K, and Z. Two subjects were able to complete 27 out of 36 trials correctly. In addition, patients reported seeing a defective array with distorted and dropout letter shapes, making it difficult to correctly identify letters. A follow-up survey was conducted with 32 patients implanted with the second-generation product, and the results showed that the number of truly effective electrodes ranged from 46 to 60 [6]. With the best achievable visual acuity of 20/1260 after implantation, patients were still not able to perform visual tasks smoothly in daily life. Beyeler et al. [37] also used a single-letter recognition test to assess the functional visual improvement after the implantation of the Argus series in four patients. The results showed that the recovery of visual acuity ranged from 20/15,887 to 20/1260 after implantation, while the patients all reported they obtained deficits in vision, resulting in dropout letter information and increased recognition time. Other trials were conducted to test the effect of Argus^®^ II implantation on their performance in motion detection, and clinical trial results showed that half of patients had improved ability to detect moving targets [38]. Abhishek et al. [39] tested the electrode and retinal gap distance using Cirrus HD-OCT software and obtained a series of clinical trial data after 1 month, 3 months, 6 months, and 1 year postoperatively. The clinical trial data showed that the distance between the electrode array and the retinal gap had an effect on the patient’s ability to complete the square localization task. The greater the distance, the weaker the light sensation produced by the stimulated electrode, while the closer the distance, the greater the light sensations that may be produced.

The IRIS prosthesis, which has 150 electrodes and is in the internal surface of the retina, was implanted in 2017. Several clinical trials tested visual tasks in 10 patients after implantation [40], and the results showed that the mean error distance was reduced from 8 to 2 in the square localization test and the mean accuracy for image recognition was improved from 45% to about 55%. However, the performance of image recognition with the device did not reach the passing line (60%), and the device had a short life span. Twenty participants with epiretinal prosthesis IMI consisting of a platinum microelectrode [41,42] stated during a follow-up period that they could perceive a weak sense of light with the prosthesis, which was not sufficient to help with activities of daily living. Retinal detachment occurred in some patients during the 3-month follow-up period.

### 2.2. Subretinal Prostheses

Alpha-AMS is the representative of the subretinal prostheses, an implant containing an array of 1500 active microphotodiodes implanted subretinally. Katarina et al. [43] reported the performance of three patients with Alpha-AMS subretinal implants on a 26-letter recognition test in which the patients were able to recognize only five letters, T, V, L, I, and O, when each letter was displayed individually. Among them, some investigators summarized the visual acuity of patients after the implantation of Alpha-AMS [44] and showed that the optimal level of visual acuity that the patients could achieve was 20/546. Zrenner et al. [45] evaluated the recovery of functional visual acuity in patients after the implantation. Two patients were unable to identify the Landolt C ring and individual letters, and only one patient was able to identify individual letters such as L, T, and Z for a maximum of 60 s. Two patients were able to distinguish between the different positions of the letter “U” opening and achieved 73% and 88% correct response rates. Meanwhile, some patients recognized individual letters for more than 40 s after implantation, as in [32]. PRIMA [47,49], a prosthetic device with 378 electrodes, was shown to have an implantation life of up to 3 years in animals [50]. In 2018 [51], it was successfully implanted in three subjects. The follow-up comparison showed that the three patients with subretinal implantation sites achieved visual acuity between 20/460 and 20/550, whereas the two patients with suprachoroidal implantation sites achieved 20/800, indicating the subretinal implantation site was optimal. However, the optimal visual acuity after implantation was far below normal vision, and the patients still had difficulty performing visual tasks in daily life. From the above clinical results, it can be concluded that subretinal and epiretinal prostheses can both elicit a kind of light sensation called “phosphene”. Comparing the signal processing method, the former used an extra-ocular information processor, while the latter used the processing method closest to that of natural vision. From the perspective of implantation risk, the latter was less damaging to the retina, while the small space of the former poses a greater challenge for electrode encapsulation and design.

Suprachoroidal prostheses are also considered to be a type of retinal prosthesis. Fujikado et al. [52] implanted a developed suprachoroidal prosthesis with 49 electrodes into three patients with RP. For one year after surgery, all patients were tested daily at home, such as the ability to view white lines on a black background, square positioning, walking along a line, and differentiating between dishes and chopsticks, to assess the effectiveness of the suprachoroidal prosthesis in improving patients’ functional vision. Two patients with RP reported that the stimulation of the electrodes did not produce corresponding phosphenes in their expected locations [53] and that the viewed phosphene array had distortions. Mathew et al. [56,58] performed square localization (SL) testing and functional visual acuity assessment in four patients with advanced RP and four with advanced AMD after the implantation of the Bionic Eye choroidal implant with 44 electrodes. The results showed that the mean pointing error in SL decreased from 27.7 ± 8.7° to 10.3 ± 3.3° in the four RP patients who used the device, and the mean success rate for patients completing the task was lower than 40% in the four AMD patients who performed the search for objects on the table test.

### 2.3. Visual Cortex Prostheses

**Figure 3:**
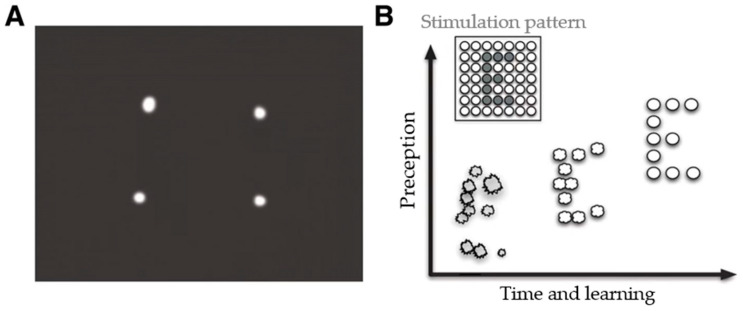
Schematic diagram of poor phosphenes induced by CORTIVIS electrode implantation. (**A**) Simultaneous stimulation of four electrodes arranged in a square may produce the perception; (**B**) Immediately after implantation, the induced phosphenes may cause poor perception of objects, such as the letter “E” in the figure. However, appropriate learning and rehabilitation strategies can help to improve the poor perception (adapted from [66]).

As early as 1976, research began on the ICVP visual cortex prosthesis project, and later ICVP used a wireless floating microelectrode array (WFMA) to replace earlier implantation kits that used a large number of wires to connect electrodes and to reduce the cost and risk of the procedure. Brindley et al. [63] implanted a completely blind patient to perform a word reading test, and the best level the patient could achieve was 8.5 characters/min. Fernández et al. [18] implanted a CORTIVIS consisting of 96 electrodes in the visual cortex of a totally blind patient for 6 months, during which the patient was given a letter recognition test. The results showed that the patient was able to recognize only five letters out of 26: I, L, C, V, and O. Another CORTIVIS prosthesis [64] produced vision by evoking 100 microelectrodes. Patients described the evoked phosphenes as flickering, colored, or colorless pinpoint stars that dropped out and were distorted during the single-letter recognition test. The irregularities are shown in Figure 3. Chen et al. [65] implanted 1024 electrodes into the visual cortexes of monkeys, and the monkeys were able to recognize simple shapes or letters after evoking the electrodes. Because the visual cortex is located in the occipital cortex of the brain, far from the center of the human visual field, this is more risky to accomplish surgically, and there have been fewer clinical trials of visual cortex prostheses.

Of the several abovementioned prosthetic devices that have entered the clinic or ended that phase, researchers have assessed the functional visual acuity of patients after wearing the prosthesis through different visual tasks. The greatest number of tests were required for single-letter recognition, square positioning, etc., but patients did not perform well on these simple tasks, such as the recognition of single letters in more than 40 s with an implant containing 60 implanted electrodes [32] and 1500 diode arrays [45]. Additionally, most of the implant recipients reported that the evoked electrodes produced phosphene points with dropouts and distorted arrays, which still bring inconvenience in achieving daily visual tasks. The researchers in the field of prostheses have started to look for relevant optimization solutions to further improve the limited visual perception.

## 3. Optimization of Information Processing in Visual Prosthetics

It is important to provide better visual perception to the patient as researchers look for factors that influence the visual perception of the implant recipient, such as the material of the electrode and the density of array [17], the stimulation parameters of the electrode [67,68,69], the distance between the electrode and the implantation site [70], and others. Seung et al. [71] used a liquid crystal polymer (LCP) to fabricate a smoothly rounded and flexible structured electrode and implanted it into the choroid of rabbit eyes, which showed that this electrode was safe and stable and could be effectively used for retinal implants. The Argus II increased the number of electrodes from 16 to 60, provided nearly four times the resolution to the patient, and was theoretically capable of providing significantly more visual information than the first generation. The characteristics of the phosphenes are influenced by adjusting the electrode stimulation parameters, such as synchronous pulses affecting the brightness and shape of phosphenes [72], producing a higher level of visual perception than asynchronous stimulation, and the degree of influence is also closely related to the configuration and location of the implanted electrodes [73]. Rebecca et al. [74] used electrodes made of activated iridium oxide (AIROF) to maintain anodic potential bias during interpulse intervals, which could satisfy the charge level required for neural stimulation and reduce electrode polarization. To avoid the phenomenon of phosphenes lasting less than half a second due to the desensitization of retinal ganglion cells, chenais et al. [75] proposed a more natural stimulation strategy based on the temporal modulation of electrical pulses, which was effectively validated on experimental mice with a duration of 4.2 s.

Hardware upgrades, such as increasing the number of electrodes, are necessary for visual prosthetic devices. However, there are currently more difficulties in practice. Therefore, researchers are looking for image processing strategies to optimize the visual information at a low resolution so that implant recipients can better understand the artificial vision available with current prosthesis devices. The optimization of the image processing strategy mainly uses effective techniques in computer vision to extract useful information and to propose certain expression methods according to different visual tasks. Finally, more useful visual information is provided to the recipients. Depending on the target in the assessment of functional vision with a visual prosthesis, widely studied visual tasks include face recognition, letter recognition, and object recognition.

### 3.1. The Optimization Strategy of Face Recognition

Human beings socially communicate with people very frequently in daily life, so learning how to improve face recognition through image processing is one of the important directions in prosthesis research. The related studies conducted a recognition task with either unfamiliar or familiar faces. Boyle et al. [23] designed six processing schemes by image enlargement for subjects to choose the best scheme for face recognition under prosthetic vision. The results showed that the image optimization based on the magnification window of saliency detection was the most chosen, and thus it was considered as the most effective one. Wang et al. [76] proposed three face detection strategies for investigating the appropriate regions for face recognition under artificial prosthetic vision. The first one was to detect faces with the Viola–Jones face detection technique (VJFR) and box out face regions; the second one was to extract face regions according to statistical face ratios (SFR) based on the results of VJFR; the third one was to use the matting face region (MFR) depending on the detection of the previous two methods. Subjects achieved the best recognition accuracy of 67.22 ± 14.45% with the low resolution of the three methods. In the meantime, the experimental results indicated that hair was important for familiar face recognition at a low resolution.

**Figure 4:**
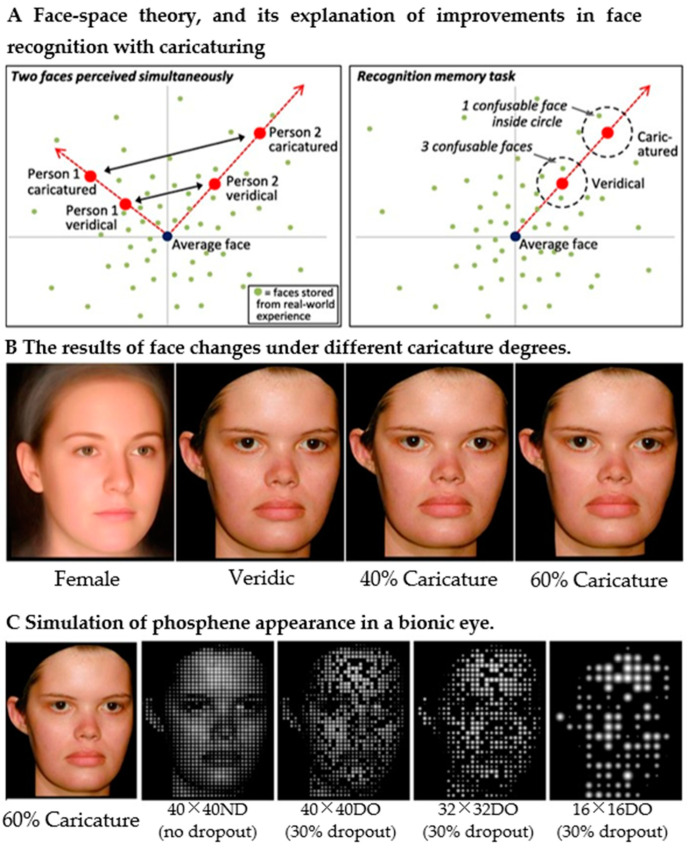
Schematic diagram of caricatured human face. (**A**) How faces are represented in the brain, and how this explains improved performance with caricaturing. The dimensions coded on the axes remain unknown, so they might represent the width of the face or other variables. (**B**) Examples of caricatured faces. Facial features are altered, e.g., the higher the degree of caricature, the thicker the lips become. (**C**) The leftmost is the face after 60% caricature. The three images from right to left are phosphene images with random dropout at different resolutions (adapted from [78]).

Interior feature extraction is particularly important in familiar face recognition because interior features (e.g., glasses, nose, and mouth region) can help subjects identify familiar faces. Rollend et al. [77] also proposed an image enhancement method using efficient local binary pattern (LBP) features to detect faces when the detected face intersects the an implanted field of view, segmenting the area around the face with an ellipse, performing face contrast enhancement by histogram equalization, and achieving real-time face detection at a low resolution. Moreover, to highlight interior features, Jessica et al. [78] caricatured the face image to exaggerate the identity information in both familiar and unfamiliar face recognition. The average faces of females and males were calculated from the locations of the marked face attributes. By exaggerating the distance between attributes such as the target face’s and the average face’s lips, people with thick lips were caricatured so that the lips became thicker. At a resolution of 40 × 40 and a dropout rate of 30%, the average face recognition accuracy of the subjects improved from 55% to 65%, exceeding the passing level. The schematic illustration of the principle is shown in Figure 4, A, B and C in Figure 4 are the results of the face processing. To further reduce the difficulty of face recognition, Zhao et al. [79] proposed a FaceNet-based strategy to transform and replace complex face information into simple Chinese characters (surnames) in real time, resulting in recognition accuracy values of 77.37% and above, providing a new possible direction for improving face recognition in the field of prosthetics. Chang et al. [80] combined Sobel edge detection and contrast enhancement techniques to highlight interior features of familiar faces. The proposed contrast enhancement was a novel histogram equalization technique that adjusted the input histogram by adaptively changing parameters to enhance the image naturally. The face images selected in the experiments were all familiar faces for the subjects. The results showed that the subjects’ face recognition accuracy reached 27 ± 12.96%, 56.43 ± 17.54%, and 84.05 ± 11.23% for the three resolutions (8 × 8, 12 × 12, and 16 × 16), respectively, while the subjects’ average response times for recognizing facial images were 3.21 ± 0.68 s, 2.73 s, and 1.93 ± 0.53 s, respectively. Recently, Xia et al. [81] proposed an F2Pnet for translating faces into pixelated faces, and 14 subjects were recruited for tests of face recognition. The training dataset was AIRS-PFD, and the results showed that mean individual identifiability values were 58% with pixelated faces and 46% with reduced resolution and display degradation (30%).

Methods such as image enhancement and edge detection for interior face features have been shown to be helpful in improving face recognition under prosthetic vision, and some methods have been applied in prosthetic devices [82]. However, the study of image optimization algorithms for face recognition under irregular artificial prosthesis vision were relatively small, and the resolution used in the relevant studies that have been conducted was high, much higher than the number of electrodes in the more widely implanted prosthetic devices. The performance of the image optimization for face recognition deserves further research by taking into account the irregularities in real artificial vision.

### 3.2. The Optimization Strategy for Character Recognition

**Figure 5:**
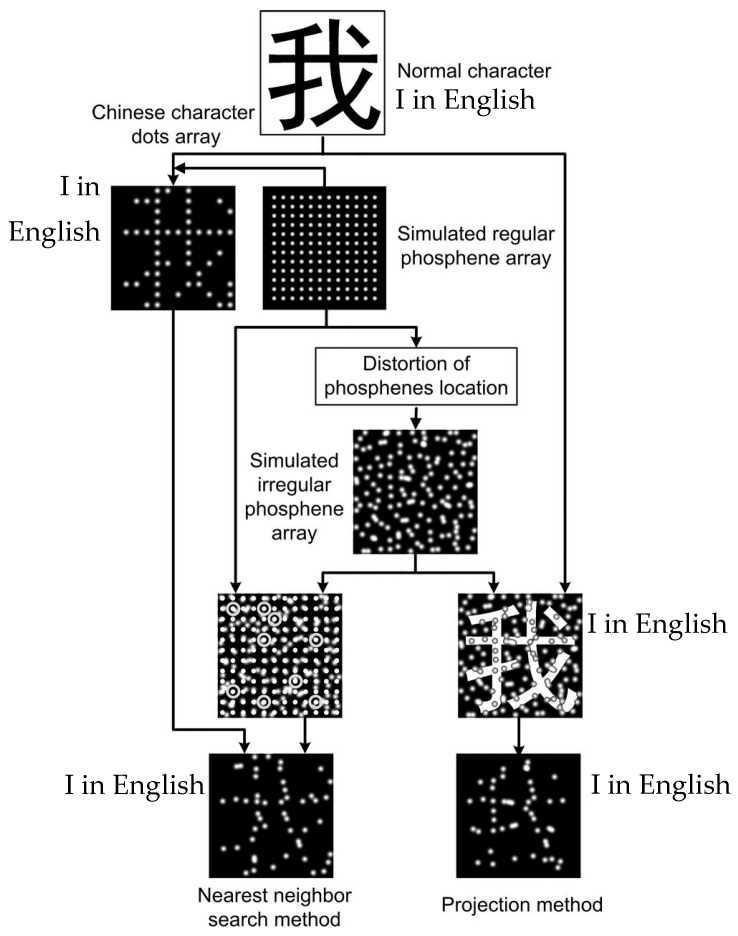
Two optimization strategy procedures (cited from [87]).

**Figure 6:**
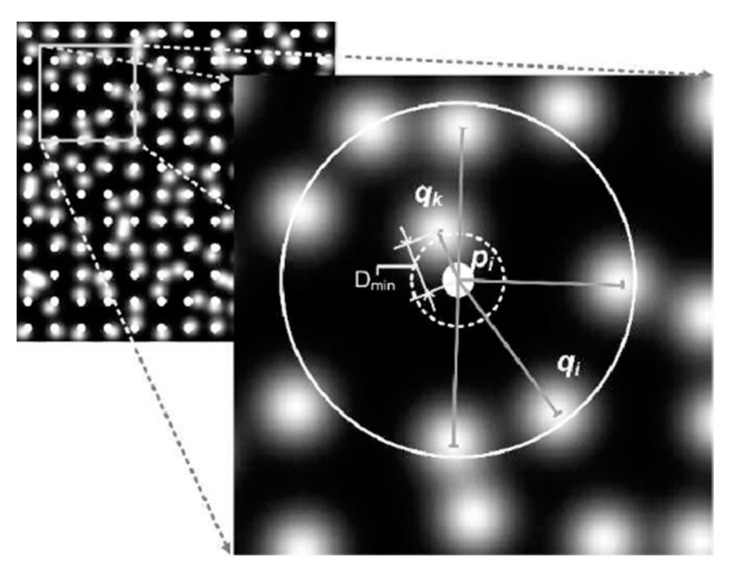
Principle of optimization of Chinese characters for nearest neighbor search. $p_{i}$ is the ideal location for the induced phosphene point of view, $q_{i}$ is the location of the actual induced phosphene, $q_{k}$ is the location of the phosphene induced by another electrode, $D_{\min}$ represents the shortest distance (cited from [87]).

Character recognition has likewise received much attention, as an important direction in prosthesis research. Early studies focused on the effects of phosphene properties, such as dot size and number, on character recognition [11,83,84,85]. Some of the work utilized image processing methods, such as Fu et al. [86], who processed images with cropping and segmentation. Considering the presence of dropout phosphenes and array distortions, some researchers have improved the adverse effects of such irregularities through image processing. Dai et al. [26] proposed two correction methods, including weighted nearest neighbor search (NNS) and expansion based on image morphology, to improve the recognition of Chinese characters under irregular phosphene arrays. The results demonstrated that the average accuracy after using the correction was more than 80% when the index of array irregularity reached 0.4. Based on this work, Lu et al. [87] optimized the NNS and further proposed a projection method to improve the reading ability of subjects with irregular phosphene arrays, and its specific processing flow is shown in Figure 5. In Lu’s study, the NNS found the evoked irregular phosphene array for the nearest phosphene dot, $q_{k}$, in a circle that centered on the point $p_{i}$ in the ideal regular phosphene array. The schematic diagram is shown in Figure 6, where the observed $q_{k}$ replaces the $q_{i}$ to express visual information. Projection refers to superimposing normal characters on a phosphene array of the same size and pixelating the strokes over the viable phosphenes to finally generate the corresponding pixelated character results.

By the experimental results, the accuracy of Chinese character recognition under both strategies is higher than that before optimization, and the effect is better under the nearest neighbor search optimization strategy. The results also indicated that, in the NNS, the larger the search range selected by the nearest neighbor search strategy, the higher the subjects’ recognition accuracy. The accuracy reached or exceeded 69.4 ± 3.4% when the search range reached or exceeded 0.6 times the adjacent phosphene point spacing. This is due to the fact that the NNS method complements the absent features influenced by the distortion and dropout of character strokes while preserving the structure of Chinese characters.

Kiral-Kornek et al. [88] proposed to extract edge orientation information encoded as a directional elliptical phosphene to improve letter recognition performance under prosthetic vision. The results showed that, considering a dropout rate of 50%, the subjects achieved 65% recognition accuracy using the directional phosphene strategy, significantly higher than the 47% recognition accuracy under the uniform stimulation strategy. Hyun et al. [89] investigated the effects of image presentation methods on character recognition at different stimulus frequencies. If the electrode stimulation frequency is too fast, it may cause the subject to see multiple phosphenes or even a large phosphene directly occupying the entire visual field. At the two resolutions of 6 × 6 and 10 × 10, Hyun et al. used two methods of pixelization for Korean and English letters, the static pixelization method and the spatiotemporal pixelization method (SP). The SP method means that the original image was downsampled with a block-averaging algorithm with four times the spatial resolution of pixelation, while the block-averaged image was subsampled to four different low-resolution images. A two-dimensional Gaussian function was convolved on each subsampled image to generate four different phosphene results, which were presented to the subjects at different stimulus frame rates. The strategy of spatiotemporal pixelation significantly improved the recognition accuracy from a failing grade to 80%. This method of sequential stimulation of subsampled images “splits” the stroke structure into four parts and takes advantage of the characteristics of the human brain’s short-term memory to achieve character recognition.

Character recognition is an important part of life, and researchers have used computer vision image processing methods to assist visually impaired people with character recognition. Character/letter recognition is not as difficult and commonly does not require high visual acuity compared to face and object recognition. Likewise, in prosthetic vision, characters/letters do not need many phosphenes to convey information, using mostly simple preprocessing methods such as binarization. During the clinical trial phase, the implant recipients were able to perform character recognition faster or better with a short training period compared to the other visual tasks tested [43]. To reduce the adverse effects of irregular artificial vision on reading or daily word communication, researchers have proposed several array optimization methods, which have been validated in Chinese character recognition under simulated artificial vision. After irregularity optimization under simulated prosthetic vision, subjects have achieved more than 80% accurate recognition rates [26]. Even in the recognition of Chinese characters with complex strokes, there was a large improvement in recognition rate with optimized correction. Therefore, in recent years, there were less studies on character recognition and its information optimization methods. On the other hand, the role of these correction methods in other language characters should be further investigated in the future.

### 3.3. The Optimization Strategy of Object Recognition

Similar to the studies of face recognition, research on object recognition in this field aims to extract and enhance useful information from the low-resolution artificial vision field to help the implant recipients to obtain better visual perception and object recognition ability. Li et al. proposed a top-down model for global contrast saliency detection [90], which detects and extracts the most conspicuous objects in the scene in real time by combining color and intensity differences. A set of visual tasks was designed with simulated ideal artificial vision. Subjects were asked to find the target object at a distance of 2 m. The average time to complete the task before using the optimized strategy was around 62 s. Afterwards, the average time was around 42 s using the optimized strategy. In order to better assess the effect of the optimization strategy on daily life, a second eye–hand coordination task was designed in which subjects were asked to find two target objects among the four objects in front of them and complete the corresponding actions. Meanwhile, the mean rate of correctly completed tasks (PC) increased from 62.85 ± 1.54% to 84.72 ± 1.41%, and the mean completion time (CT) decreased from 49.5 ± 3.76 s to 40.73 ± 2.1 s. Analyzing the experimental data together, the mean PC and CT with both types of vision tasks verify the effectiveness of the optimization strategy. Meanwhile, the mean head motion of the subjects decreased from 939.31 ± 38.38° to 575.70 ± 38.53°, indicating the searching scope was significantly reduced and the ability to perceive was improved. A year later, Li et al. [91] used another bottom-up saliency detection model, graph-based visual saliency (GBVS), combined with edge extraction to help locate foreground regions, which could also help the recognition of one or two objects of interest at an ideal low resolution.

**Figure 7:**
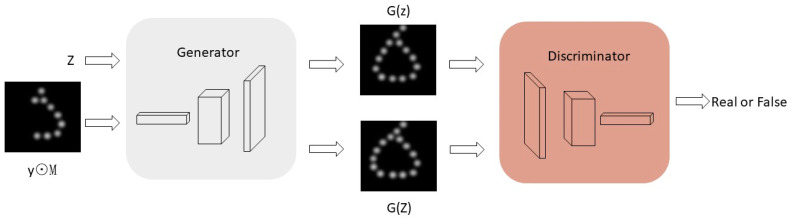
The principle of optical illusion point of view supplement. z is arbitrary Gaussian noise; M is the dropout part; in the binary mask, 0 indicates the dropout position of the phosphenes, and 1 indicates the retained phosphene position; y is the input image; y⊙M is the image of ideal phosphenes. G(z) is a mapping close to the real generated image obtained from the Gaussian noise z. Z is the optimal solution. G(Z) is the most suitable mapping to represent the dropout part (adapted from [93]).

Considering the deficit in real artificial vision, Li et al. [92,93] utilized a generative adversarial network (GAN) model, which has had remarkable success in the field of image inpainting, to compensate for the absence of phosphene points. The Pix2pix GAN was used to learn the mapping relationship between RGB images and pixelated results with a generator and a discriminator, which generates the pixelated results with additive phosphenes points close to the real one. The principle of the model is shown in Figure 7, and the calculation is shown in Equation (1).

(1) $$pixel_{reconstructed}=y⊙M+(1-M)⊙G(Z)$$

where the input binary mask is denoted by M; the dropout image of the input phosphene point is denoted by y; G(Z) is the mapping suitable for representing the missing parts, and ⊙ is the Hadamard product.

Inputting arbitrary Gaussian noise, *z*, into the generator, the image features of any simulated ideal phosphenes were learned to obtain a mapping close to the real generated image, G(z), where the input to the generator was any image, y, with dropout phosphenes. The Hadamard product of y and the binary mask of dropout part M were calculated to obtain the image y⊙M of the no dropout part. Simultaneously, the discriminator determined the difference between the input image, y, and the real image using difference back-propagation and adversarial training to reconstruct the dropout part of the image, y, to obtain the optimal result, G(Z), for which the generator used previously learned features, where the optimal solution, Z, was obtained by back-propagating the updated generator parameters in the process of minimizing the global loss. Then, the no-dropout part, y⊙M, and the optimal result of generating the dropout part, G(Z), were summed to obtain the final complemented complete image, $pixel_{reconstructed}$. Subjects were asked to answer verbally the identity of the object appearing on the screen within 10 s at a distance of 35–40 cm from the display. The test results showed that the average recognition accuracy of subjects ranged from 35.0 ± 4.3% to 60.0 ± 6.1%, and the accuracy improved to 80.3 ± 7.7% after using the phosphene point addition optimization strategy. The model decreased the difficulty for subjects in the recognition process, and they could recognize most of the pixelated objects.

Aiding the visual prosthetic wearer in subsequent daily perceptual tasks through scene understanding, Melani et al. [94,95] used a strategy combining structural informative edges (SIE) and objects mask segmentation (OMS) to help identify objects and rooms. Among them, the objects in the indoor scene were highlighted with instance segmentation to reduce the interference of the background, the structural information in the scene was extracted with semantic segmentation, and the edge information in the scene was extracted with Canny edge detection. Object recognition and scene recognition tests were conducted to simulate the daily life of subjects in a 20° viewing angle with a 32 × 32 resolution. In the object recognition task in direct low pixelation, the subjects’ correct object recognition rate was 36.83%, compared to 62.78% with the SIE-OMS strategy. The recognition success rate for the scene recognition task with the same strategy was significantly higher than that with direct low pixelation and edge detection. To reduce the difficulty of recognition when multiple target objects within the field of view appear to overlap, Jiang et al. [96] proposed a hierarchical method to assign different levels of grayscale values to multiple targets according to an object’s location, size, and degree of importance in the scene based on the segmentation of multiple targets with Mask RCNN. Ultimately, the subjects achieved a average task completion rates of 87.08 ± 1.92% on the test describing the number of multiple objects in the scene and 60.31 ± 1.99% on the test describing the content of the scene. Considering the depth information of objects, David et al. [97] proposed an InI-based object segmentation model that extracts objects from the scene based on their depth information, derived from the distance from the camera (simulating a human eye), pixelates the extracted objects to improve the clarity of objects in the vision, and reduces some of the distracting spatial and temporal effects. Other scholars have conducted studies under other image acquisition methods, such as Dagnelie et al. [98], who used infrared enhancement to help subjects with cup recognition. Again, using infrared images, Liang et al. [99] proposed an infrared image enhancement algorithm with an improved SAPHE algorithm to enhance image contrast and highlight edge contours to help highlight edges for object recognition. The experimental results showed that the subjects achieved an average recognition accuracy of 86.24 ± 1.88% under infrared mode processing, which was higher than the 64.55 ± 3.34% with direct low-resolution pixels. The depth information of images plays an important role in obstacle avoidance navigation tasks. Alejandro et al. [100] used depth information to detect obstacles while guiding the walking direction with a chessboard grid pattern. To make better use of the depth information in the scene, Rasla et al. [101] proposed a scene simplification strategy based on depth estimation and semantic edge detection using a neurobiologically inspired bionic visual computational model to simulate obstacle avoidance tests, where depth estimation was implemented by a self-supervised monocular depth estimation model using monodepth2 to predict the relative depth map of the pixels in each frame. Simplifying the scenario by semantic edge detection, the success rate for subjects with zero collision obstacles was above 80% in the obstacle avoidance test under simulated prosthetic vision using the combination of depth information and edges.

The method based on saliency detection and segmentation, which were widely used in the field of visual prosthesis, detects the most salient object (objects) in the field of view, removes complex backgrounds, and satisfies the requirement of providing limited information in a low resolution. Some scholars have carried out studies on the methods of depth in detecting useful information related to the task. Few researchers have focused on the presence of deficits in artificial vision and proposed correction methods for object recognition. Further research should consider the proposed optimization method under more irregular artificial vision conditions, such as distortion.

### 3.4. Summaries of Optimization of Information Processing

Since the research on the optimal processing of visual information was carried out, different computer vision methods were applied to the image processing stage, and the subjects showed improvements in their performance on different visual tasks. Table 2 summarizes the image processing optimization methods in three vision tasks with or without considering the phosphene point dropout or phosphenes array distortion as well as the optimization and final test results.

In the simulation experiments conducted with several reviewed and analyzed image processing optimization strategies, the selected subjects were normal-sighted and unfamiliar with artificial vision to avoid learning effects in psychophysical experiments. The datasets used in the experimental process were mostly created by the researchers themselves. Among them, a few studies used public datasets, such as the public dataset of indoor scenes [102] used in the study of Melani et al. [94,95] and the ETH-80 adopted by Li’s work [92]. In the face and character recognition, some well-known datasets were also utilized, such as AIRS-PFD in Xia’s work [81] and the standard MNREAD reading test in the work of Fu et al. [86]. Some others captured images directly from the camera in real time as experimental images. However, without a public dataset, it is difficult to generalize the researchers’ experimental results. Preprocessing was sometimes applied by common methods or computer vision techniques, such as the work of Boyle et al. [23] cropping images to make them fit the window size. Wang et al. [76] used noise reduction for face recognition, and the work of Chang et al. [80] extracted the edges of faces in images with Canny operators. Before the layering and optimizing of object information, Li et al. [96] utilized Mask-RCNN to obtain the mask of multiple object instance, which could be regarded as processing. Meanwhile, certain image processing optimization strategies have high hardware requirements, and computing devices with GPUs are essential to the implementation of the algorithms in real time. The above image processing optimization strategies applied to different visual tasks brought better visual perception to the subjects to some extent, and the results of the simulation experiments in each study mostly quantitatively assessed the effectiveness of these image processing strategies. However, the majority of them are based on simulation studies under ideal arrays [21,22,90,91,96,99,103,104,105,106,107,108], except for [26,78,81,87,92], which consider irregularities in real artificial vision.

## 4. Discussion

Visual prosthetics have provided an important research direction for repairing the visual perception of the visually impaired and have been promoted with certain clinical applications. Visual prostheses are not a fundamental solution to the visually impaired, but they provide the opportunity for the visually impaired to improve their ability to perform functional visual tasks. This paper reviews the visual perceptual ability of implant recipients in clinical trials and studies of image processing optimization in the field of simulated prosthetic vision. Although the experimental results from many studies were promising, some problems still need to be solved. Fewer prosthetic devices have entered the clinical phase, and some are implanted far from the center of the visual field and are not well-perceived by the wearer. Implantation into the patient’s eye can take a long time [99,109,110,111,112,113] and carries surgical risks. The number of electrodes in these devices is limited, and there are irregularities in the induced phosphenes. The more widely worn Argus II has an external image processing unit that includes edge detection and enhancement technology. However, these methods are simpler and provide global important information to the wearer, which is less related to the visual tasks. While image processing algorithm research is spreading in the field of visual prosthesis applications, some issues are worth considering. Most of the image processing algorithms investigated by researchers are image optimization methods used on single vision tasks in static images. However, in real life, people typically perform two or more visual tasks at the same time, such as putting on clothes, where people may perform object detection and eye–hand coordination simultaneously. The multitask requires image processing methods that guarantee good performance while ensuring real-time implementation. However, few models in the current research can meet such requirements. Meanwhile, the dropout of phosphene points and the distortion of phosphene arrays in artificial vision were less considered. Additionally, the simulated phosphenes are mostly colorless. However, clinically evoked ones are colorful, such as yellow, red, and orange [65,114,115]. Recently, Vernon et al. [116] proposed a hybrid stimulus model that can provide color information without reducing spatial resolution. The understanding of colored phosphene vision will help improve research on visual function restoration for artificial prosthesis wearers. Some researchers have now made improvements to electrode implantation by proposing an array in a honeycomb configuration [117]. This unique configuration shape offers great possibilities for improving the spatial resolution of the visual prosthesis. On the basis of existing image optimization research, future research will focus on irregular array optimization under different image categories, and increasing the color information of phosphenes should be carried out in the process of exploring the improvement in the resolution of visual prostheses.

## 5. Conclusions

In studies of the optimization of information processing, most show that computer vision can be used to improve the visual functions of wearers, such as object recognition, face recognition, and character recognition. Future visual prosthesis devices may have smaller implanted electrodes, allowing for the implantation of higher density microelectrode arrays. However, as the density of electrode arrays increases, it may not always produce the expected high-resolution artificial visual information and may bring such phenomena as virtual electrodes and increase the risk of tissue damage and the cost of implantation. These issues indicate that the growth in the number of implanted electrodes will be limited in the near future. With the growing development in the field of artificial intelligence, more accurate and efficient image detection and segmentation techniques are ongoing, offering the possibility of improving image processing modules in prosthetic devices to optimize artificial vision. It is believed that along with the improvement in visual prosthesis device hardware and the application of computer vision, the two complement each other to optimize the elicited vision of artificial visual prostheses, bringing the hope of “seeing” to prosthetic wearers.

## Figures and Tables

**Table 2:** Image processing optimization on visual prostheses.

Visual Tasks	Optimization Methods	Array Distortion	Distortion	Dataset	Evaluation Indicators	Results
			Optimization			
	Significance	no	no	self-construction		Subjects selected the
	amplification				Subject	significance amplification
	window				preference	window as the most
	[23]					helpful method.
		no	no	self-construction	Recognition accuracy	The recognition accuracy of
	VJFR;					VJFR-ROI, SFR-ROI, and
	SFR;					MFR-ROI were
						52.78 ± 18.52%, 62.78 ± 14.83%
	MFR					and 67.22 ± 14.45%
	[76]					respectively
	Histogram	no	no	self-construction		real-time
						face detection
	equalization				Algorithm	at low resolution (30 fps)
	enhancement				runtime	
	[77]	yes	yes	26 faces		Correct recognition rates of
Face	Caricatured human				Recognition	53% and 65% were
Recognition	face [78]				accuracy	obtained with old faces and
						new faces, respectively.
	FaceNet [79]	no	no	self-construction		The average face
					Recognition	recognition accuracy
					accuracy	obtained by the subjects
						reached over 77.37%.
		no	no	self-construction		The average recognition
						accuracy at 8 × 8, 12 × 12, and
						16 × 16 resolutions were
	Sobel edge				Recognition	27 ± 12.96%, 56.43 ± 17.54%,
	detection and				accuracy,	and 84.05 ± 11.23%,
	contrast				response time	respectively; the average
	enhancement					response times were
	techniques [80]					3.21 ± 0.68 s, 0.73 s, and
						1.93 ± 0.53 s.
	F2Pnet [81]	yes	no	AIRS-PFD		Mean individual
					Individual	identifiability of 46% at a
					identifiability	low resolution with
						dropout
		yes	yes			The irregularity index
				Commonly Used		reached 0.4, and the
	NNS and			Chinese	Recognition	average recognition
	expansion			Character	accuracy	accuracy of the subjects
	method [26]			Database		after using the correction
						method was over 80%.
		no	no	Standardized	Reading speed	The reading speeds of the
				MNREAD		subjects using 6 × 6 and 8 × 8
	Threshold judgment			reading test		resolutions reached 15
	[86]			provided by		words/min and 30
				Dr. G.E. Legge		words/min.
		yes	yes	Commonly used		
Character				modern Chinese		The recognition accuracy of
Recognition	Projection and			characters (the	Recognition	the subjects using the NNS
	NNS [87]			first 500 in the	accuracy	method exceeded 68%.
				statistical table)		
		yes	no	N, H, R, S		The average recognition
	Directed				Recognition	accuracy of the subjects
	phosphenes [88]				accuracy	was 65%.
	SP [89]	no	no			After SP, the character
				26 English letters	Recognition	recognition accuracy of the
				40 Korean letters	accuracy	subjects broke the passing
						line (60%).
	Checkerboard-style	no	no		NA *	NA *
	phosphene guide			RGB-D camera		
	walking [100]			capture		
			no	self-construction	Percentage of	The mean PC of subjects in
					correctly	single task was
					completed	88.72 ± 1.41%, mean CT was
	Top-down global				tasks (PC),	41.76 ± 2.9s, and mean
	contrast				completion	HMID was 575.70 ± 38.53°;
	significance				time (CT),	the mean PC of subjects in
	detection [90]				head	multitask was 84.72 ± 1.41%,
					movements in	mean CT was 40.73 ± 2.1 s,
					degrees	and mean HMID was
					(HMID)	487.38 ± 14.71°.
		no	no	self-construction		The average recognition
						accuracy of the subjects
	GBVS and edge				Recognition	was 70.63 ± 7.59% for single
	detection [91]				accuracy	object recognition and
						75.31 ± 11.40% for
						double-target recognition.
	Generating an	yes	yes	ETH-80		The subjects were able to
Object	additive model for				Recognition	accomplish an average
Recognition	adversarial				accuracy	recognition accuracy of
	networks [92,93]					80.3 ± 7.7% for all objects.
	SIE-OMS [94,95]	no	no			Object recognition correct
				Public	Recognition	rate reached 62.78%; room
				Indoor scenes	accuracy	recognition correct rate
				dataset [102]		reached 70.33%.
		no	no	self-construction		Subjects achieved a mean
						PC of 87.08 ± 1.92% in the
					Percentage of	object test task in the
	Mask-RCNN layers				correctly	description scene and a
	[96]				completed	mean PC of 60.31 ± 1.99% in
					tasks (PC)	the description scene
						content test.
	InI-based object	yes	no	self-construction	NA *	NA *
	segmentation [97]					
	Improved SAPHE	no	no	Captured directly	Recognition	The average RA of subjects
	algorithm [99]			with the camera	accuracy (RA)	was 86.24 ± 1.88%.
		no	no	self-construction	Success rate	The success rate for
	Depth and edge					subjects with depth and
	combinations [101]					edge was over 80%.

* NA stands for No Assessment Indicators and Experimental Assessment Results.

## Data Availability

Not applicable.
